# Experimental *Mycobacterium bovis* infection in three white rhinoceroses (*Ceratotherium simum*): Susceptibility, clinical and anatomical pathology

**DOI:** 10.1371/journal.pone.0179943

**Published:** 2017-07-07

**Authors:** Anita L. Michel, Emily P. Lane, Lin-Mari de Klerk-Lorist, Markus Hofmeyr, Elisabeth M. D. L. van der Heijden, Louise Botha, Paul van Helden, Michele Miller, Peter Buss

**Affiliations:** 1 Department of Veterinary Tropical Diseases, Bovine Tuberculosis and Brucellosis Research Programme, Faculty of Veterinary Science, University of Pretoria, Onderstepoort, South Africa; 2 National Zoological Gardens of South Africa, Pretoria, South Africa; 3 Department of Research and Scientific Services, National Zoological Gardens of South Africa, Pretoria, South Africa; 4 Department of Agriculture, Forestry and Fisheries, State Veterinary Office, Kruger National Park, Skukuza, South Africa; 5 Veterinary Wildlife Services, South African National Parks, Kruger National Park, Skukuza, South Africa; 6 Department of Infectious Diseases and Immunology, Faculty of Veterinary Medicine, Utrecht University, Utrecht, The Netherlands; 7 DST/NRF Centre of Excellence for Biomedical TB Research/MRC Centre for Tuberculosis Research, Division of Molecular Biology and Human Genetics, Faculty of Medicine and Health Sciences, Stellenbosch University, Cape Town, South Africa; University of Minnesota, UNITED STATES

## Abstract

Tuberculosis caused by *Mycobacterium bovis* is endemic in the African buffalo (*Syncerus caffer*) population in the Kruger National Park and other conservation areas in South Africa. The disease has been diagnosed in a total of 21 free ranging or semi-free ranging wildlife species in the country with highly variable presentations in terms of clinical signs as well as severity and distribution of tuberculous lesions. Most species are spillover or dead-end hosts without significant role in the epidemiology of the disease. White rhinoceroses (*Ceratotherium simum*) are translocated from the Kruger National Park in substantial numbers every year and a clear understanding of their risk to manifest overt tuberculosis disease and to serve as source of infection to other species is required. We report the findings of experimental infection of three white rhinoceroses with a moderately low dose of a virulent field isolate of *Mycobacterium bovis*. None of the animals developed clinical signs or disseminated disease. The susceptibility of the white rhinoceros to bovine tuberculosis was confirmed by successful experimental infection based on the ante mortem isolation of *M*. *bovis* from the respiratory tract of one rhinoceros, the presence of acid-fast organisms and necrotizing granulomatous lesions in the tracheobronchial lymph nodes and the detection of *M*. *bovis* genetic material by PCR in the lungs of two animals.

## Introduction

Bovine tuberculosis (BTB), caused by *Mycobacterium bovis (M*. *bovis)*, is a chronic, progressive debilitating disease characterized by necrotizing granulomas in the lungs and lymph nodes in many free-ranging wildlife species, with clinical signs only visible in the later stages of the disease [1]. Susceptibility to this multi-host pathogen, transmissibility and impact of the disease at population level differ vastly between species, but are largely unknown for most wildlife species [2]. In the highly gregarious African buffalo (*Syncerus caffer)*, as in domestic cattle, the disease is progressive with the formation of necrotic liquified lymph node and lung lesions that facilitate transmission either through direct contact or indirectly through the environment [3].

Tuberculosis is rare in perissodactyls [4,5]. In rhinoceroses, *M*. *bovis* infections have been occasionally reported in captive individuals [6,7], and extremely rarely in free-ranging animals. In 2009, localised incidental BTB lesions were reported in a semi-captive black rhinoceros (*Diceros bicornis minor*) in South Africa [8] and, in 2014, a semi-free ranging black rhinoceros was found dead with extensive lesions consistent with BTB from which *M*. *bovis* was isolated (M Otto, personal communication). Although these cases illustrate the potential for natural infection of rhinoceroses with this organism, no indication of clinical infection in free-ranging rhinoceroses exists and the health impact in free-ranging animals remains unknown.

The Kruger National Park (KNP) and iMfolozi/Hluhluwe Park (HiP) have large numbers of both white (*Ceratotherium simum*) and black rhinoceroses (*Diceros bicornis*) which share range and resources with BTB-infected buffalo populations, which are important maintenance hosts for this disease [9,10]. In the KNP, zonal prevalence rates in buffalo vary from ± 40% in the south to ± 5% in the north [11]. Due to an overlap in grazing and water sources frequented by both rhinoceroses and buffaloes a potential for disease transmission exists, especially in the south due to the high prevalence of BTB-infected buffaloes and high concentrations of rhinoceroses. Currently there is no indication that BTB is a significant threat to the conservation of rhinoceros populations. However, in the event that a case of *M*. *bovis* infection is diagnosed in rhinoceroses, disease control legislation may impose an accepted and fit for purpose assay to determine infection status prior to movements of rhinoceroses out of BTB-endemic areas. In the absence of a suitable test to distinguish between infected and uninfected animals, such regulatory interventions could restrict translocation of rhinoceroses, limiting efforts to increase numbers, genetic diversity, and establish new populations. Most seriously, a movement restriction would stop conservation efforts to protect white rhinoceroses from the escalating losses due to poaching.

In response to the international demand for BTB diagnostic tests in wildlife, tools such as the STAT-PAK^®^ and DPP VetTB^®^ assays [5,12,13] and especially the rhinoceros specific interferon-gamma (IFN-gamma) assay have been recently developed [14,15]. However, these tests need to be validated in known *M*. *bovis*-infected rhinoceroses before they can be accepted for diagnostic use and risk assessments without over- or under-estimating the necessity for appropriate control measures. This paper presents a prospective study designed to establish experimental infection with *M*. *bovis* in three white rhinoceroses and to determine clinical indicators and pathological characteristics of BTB in these animals. The new knowledge gained from this research is of critical importance for optimising management decisions which support the conservation of white rhinoceroses in ecosystems endemically infected with BTB. Furthermore these known infected animals enable a first evaluation of the ability of both interferon gamma assays and serological tests to detect *M*. *bovis* infection. We describe the clinical features, hematological, biochemical and anatomical pathology induced in experimental *M*. *bovis* infection in three white rhinoceroses under captive controlled conditions. We report the absence of disseminated progressive disease in *M*. *bovis*-infected white rhinoceroses and the implications of these findings.

## Materials and methods

The South African National Parks (SANParks) Animal Use and Care Committee granted permission and the Department Agriculture, Forestry and Fisheries of South Africa granted approval under Section 20 of the Animal Diseases Act to conduct this research. SANParks was granted a TOPS Standing permit by the National Department of Environmental Affairs with regard to the keeping and use of white rhinoceroses.

### Animals

Three sub-adult male white rhinoceroses (3 to 4 years of age) were captured in September 2011 in Kruger National Park (KNP), according to the Standard Operating Procedure for the Capture, Transportation and Maintenance in Holding Facilities of Wildlife (protocol approved by South African National Parks Animal Use and Care Committee). Animals were housed in dedicated rhinoceros enclosures (bomas) at the Veterinary Wildlife Services facility in Skukuza, KNP. These animals were identified as PB 1, 2 & 3. PB 3 did not adapt to captivity and was released from the bomas eleven days after capture due to ongoing anorexia. A replacement animal, PB 4, was captured and placed into the bomas two months later. Subsequently, the remaining three animals (PB1, 2 & 4) adapted to the bomas without further complications.

The bomas contained ample shade, a water source, and food offered on cement slabs. *Ad libitum* feed consisted of a 50:50 mixture of tef (*Eragrostis tef)* and high quality lucerne (*Medicargo sativa*) hay. The animals were fed in the morning and afternoon, and water troughs cleaned and filled with fresh water daily. Feces were removed daily. Animals were under the care of a boma manager and support staff. Individual animal health status was monitored daily using a standardized scoring system that included the amount of food consumed, fecal volume and consistency, and demeanor/behaviour [16]. Any injuries, illness, or abnormal behaviour observed were immediately communicated to the supervising veterinarian.

Special measures taken to ensure safety and security of staff and facility were taken as follows. A briefing regarding health and biosafety was given to all staff involved before each procedure was taken. During the experimental infection only identified veterinarians and para-veterinarians, who had been briefed about the experimental and biosafety procedures, were allowed access to the rhinoceroses inside the boma. All such personnel wore specified protective clothing (hair cover, N95 –type face masks (suitable for protecting against aerosol exposure, coverall suits, surgical gowns, gum boots, double gloves). All used consumables were sprayed with F10 solution and placed in an autoclave bag. All protective clothing is placed in an autoclave bag, closed with tape and autoclaved. Animal caretakers were issued with detailed instructions regarding the cleaning of the bomas and they received designated protective clothing including face masks, overalls and gum boots which were exclusively used for working in the rhinoceros bomas.

### Rhinoceros immobilization

Immobilization drugs were delivered remotely using 3.0 ml plastic darts with a 60 mm uncollared needle propelled by compressed air (DAN-INJECT, International S.A., Skukuza, 1350 South Africa). Etorphine (M99, Ilanco, Kempton Park, 1619 South Africa), azaperone (Stressnil, Janssen Pharmaceutical ltd., Halfway House, 1685 South Africa), and hyaluronidase (Hylase, Kyron Laboratories, Benrose, 2011 South Africa) were mixed in the dart, and butorphanol (Butorphanol, Kyron Laboratories) administered intravenously (i.v.) within 15 minutes of darting. Doses were based on standardized weight categories: 1000 to 1250 kg– 2.5 mg etorphine, 37.5 mg azaperone, 5000 i.u. hyaluronidase; or 1250 to 1500 kg– 3.0 mg etorphine, 45 mg azaperone, 5000 i.u. hyaluronidase. The butorphanol dose was calculated at 10 mg/1 mg etorphine. Rhinoceroses were loaded into a transport crate to be weighed at the end of each monthly immobilization. Rhinoceroses received naltrexone (Naltrexone, Kyron Laboratories) (40 mg /1mg etorphine i.v.) just prior to being released from the crates.

### Preparation of *Mycobacterium bovis* inoculum and experimental infection

The resident KNP field strain of *M*. *bovis*, isolated from granulomatous tissue lesions of a buffalo in KNP in 2011 and characterized as SB0121 (TB 7913) was used to infect the rhinoceroses [17]. The isolate was sub-cultured once before preparing the inoculum for intra-tracheal inoculation. The actively growing *M*. *bovis* culture was suspended in brain-heart infusion (BHI) medium and gently homogenized to disrupt bacterial clumps. The concentration was determined microscopically using a Neubauer haemocytometer and adjusted to approximately 10^4^ cfu/ml. The inoculum was prepared by adding 8 ml of phosphate buffered saline/0.05% Tween 80 to 2 ml of the *M*. *bovis* suspension of which 7 ml was used to fill the 3.4 m long endoscopic catheter and 2 ml for instillation into the rhinoceros. The remaining 1 ml was used to prepare appropriate serial dilutions for enumeration by plating followed by incubation at 37°C for 10 weeks [18]. Standard operational procedures were followed for handling of biohazardous material.

An infective dose of 2 ml *M*. *bovis* inoculum was deposited into the two mainstem bronchi by guiding a flexible endoscope to each site and injecting fluid through a catheter inserted in the working channel. Correct placement of the catheter was visualized using a video monitor.

### Sample collection and processing

All three rhinoceroses were immobilized monthly for 5 months pre-infection and 20 months post-infection to assess health status and collect samples for hematological, biochemical, and mycobacteriological analyses. Blood was collected in EDTA, lithium heparin, and serum vacutainers (Fisher Scientific, Suwanee, Georgia 30024, USA) from the radial vein. White and red blood cell counts, platelet counts, and red blood cell parameters were analyzed using an automated hematology analyzer (Vet ABC, Scil Animal Care Company, Gurnee, IL 60031, USA) as previously described [19]. Packed cell volumes (PCV) and total protein values were measured manually using a microhematocrit centrifuge and refractometer, respectively. Blood smears were stained with eosin-methylene blue (Kyro-Quick stain, Kyron Laboratories Pty. Ltd.) for manual differential counts. Sera for biochemical analyses were processed as previously described and analysed using an ABAXIS VetScan 2 with large animal rotor [20].

Immobilized rhinoceroses were placed in left lateral recumbency to perform tracheal lavage. Medetomidine (Medetomidine, Kyron Laboratories) (1000 to 1250 kg– 5mg; or 1250 to 1500 kg– 6 mg) was administered i.v. to facilitate masseter muscle relaxation. Once the mouth was opened using a modified car jack, a wooden wedge was placed between the molars for safety. The operator was then able to manually palpate the glottis and insert a disinfected equine stomach tube into the trachea. Approximately 0.23 to 0.35 ml/kg sterile saline was instilled (i.e., 350 ml/animal) and the animal was rocked from side to side. Samples were collected using a portable suction pump which aspirated fluid into a sterile canister. Samples were immediately transferred to sterile 50 ml conical tubes and placed on ice bricks until transported to the laboratory. Lavage fluid was centrifuged at 1500 x g for 10 min. The pellet was resuspended in approximately 5 ml of supernatant and aliquots frozen at -80°C until culture could be performed. None of the rhinoceros had adverse reactions to the repeated intubation and bronchial lavage as measured by boma scoring on days after the procedure was conducted.

### Post mortem examination

All three rhinoceroses were euthanized at the end of the trial according to a Standard Operating Procedure approved by SANParks Animal Use and Care Committee. The animals were immobilized in the standard manner with etorphine and azaperone, and then 160 to 200 ml of a saturated 50:50 solution of succinylcholine chloride (Suxamethonium chloride, Kyron Laboratories) and potassium chloride (Potassium chloride, Kyron Laboratories) was administered i.v. Death was confirmed by cessation of respiration, an absence of cardiac activity on auscultation and dilation of the pupils.

A full necropsy examination was conducted, sampling all organs except the spinal cord. Dedicated teams were assigned to sectioning specific organs, recording of findings and sampling for further analyses to ensure consistency of all procedures between the three rhinoceroses. Descriptions of all organs as well as major joints and body cavities were recorded in detail. In addition to any observed lesions, complete sets of tissue samples were preserved in 10% buffered formalin. Lungs were sectioned into 2-3cm wide slices, and four random samples taken from each of the left and right craniolateral (CrL), craniomedial (CrM), caudolateral (CdL) and caudomedial (CdM) lung lobes. Each lung section was closely palpated for tiny (<1mm diameter) nodules and larger lesions. Mandibular, retropharyngeal, prescapular, deep cervical, axillary, tracheo-bronchial, mediastinal, cranial and caudal sternal, pulmonary, omental, splenic, gastric, hepatic, pancreatic, colonic, mesenteric, caecal, inguinal and renal lymph nodes were sectioned into 0.2–0.4cm slices and scrutinized for possible tuberculous lesions (nodules with or without visible calcification). Lung and lymph node nodules that were large enough were divided into 3 aliquots: a sample for histology in 10% formalin and duplicate samples for culture (frozen at -20°C until processed). Lesions too small to section were serially allocated to histopathology and culture.

Inclusion of age and sex-matched healthy white rhinoceroses as controls in the trial was considered unethical. Therefore, histological lung and lymph node sections from 11 historic cases of subadult to elderly white rhinoceroses (8 males, 3 females) that had undergone necropsy in KNP for other reasons were used as controls.

### Histopathology

**Fig 1:**
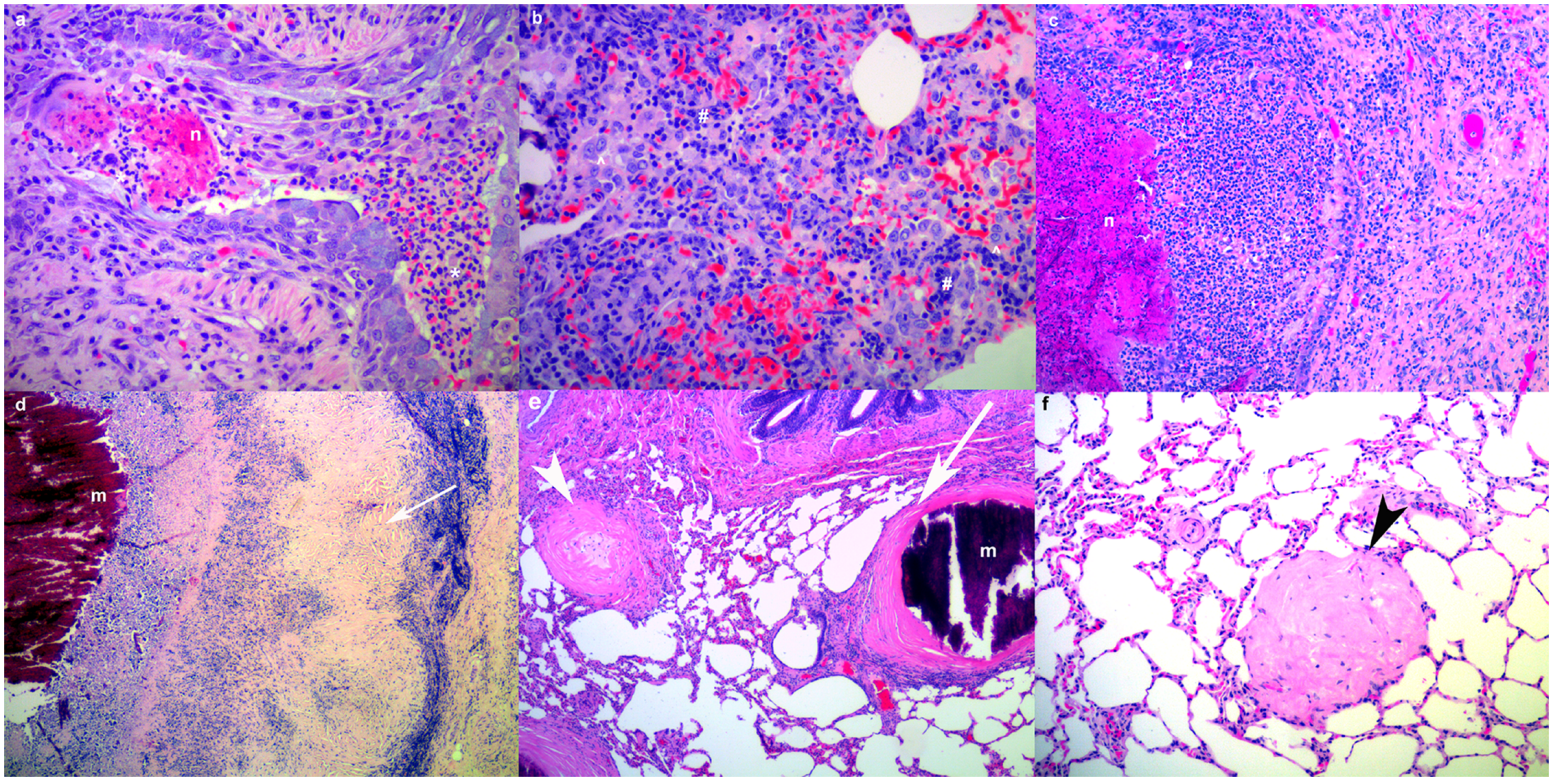
(a-f). Characterization of histological lung lesions in white rhinoceroses. (a) Acute inflammation: acute ulcerative bronchitis with necrosis (n) and neutrophils (*) in the lumen (PB2, H&E, x200). (b) Subacute inflammation: lymphoplasmacytic (#) and histiocytic (^) interstitial pneumonia (PB2, H&E, x200). (c) Subacute inflammation with necrosis: interstitial pneumonia from with central necrosis (PB2, H&E, x100). (d) Chronic pulmonary granuloma with a capsule (white arrow) and mineralized (m) cellular and inflammatory debris in the center (PB2, H&E, x40). (e) A chronic pulmonary inflammatory focus with a capsule and central mineralized material and a fibrous nodule (white arrowhead) with a few inflammatory cells in the center (PB1, H&E, x40). (f) Fibrous nodule (black arrowhead) consisting only of mature collagen (PB1, H&E, x100).

Tissue samples from experimental and control animals were processed routinely for histopathology examination [21] and stained with haematoxylin and eosin (HE). Any granulomatous lesions were also stained with Gram-Twort (GT), Ziehl-Neelsen (ZN), Gomori Methamine Silver (GMS) and periodic acid Schiff (PAS) stains for bacteria, mycobacteria, fungi or protozoa, respectively. Lung and lymph node inflammatory lesions were characterized as acute inflammation (AI, Fig 1a), subacute lymphoplasmacytic and histiocytic inflammation without granuloma formation (SI, Fig 1b), subacute inflammation without granulomas but with necrotic cellular and inflammatory debris present (Fig 1c), encapsulated chronic granulomata with variably mineralized necrotic cellular and inflammatory 1a debris in the centre (CNG, Fig 1d), encapsulated chronic inflammatory foci with mineralized material in the centre (Fig 1e), fibrous nodules with a few inflammatory cells in the centre (FIN, Fig 1e), and fibrous nodules consisting only of mature collagen (FN, Fig 1f).

### *Mycobacterium bovis* culture

Two duplicate standard sets of lymph node samples including head, chest, abdominal and carcass lymph nodes as well as samples from any macroscopic lesions were cultured from each rhinoceros.

One set of tissue samples (a total of 30 individual or pooled samples) was cultured on Löwenstein-Jensen (LJ) medium in a BSL2+ laboratory at the Faculty of Veterinary Science, University of Pretoria. Briefly, tissue samples were homogenized using Kinematica dispersing aggregates fitted into 50 ml screw cap tubes. Homogenates were split in two aliquots and decontaminated with a final concentration of 2% sodium hydroxide and 1% hydrochloric acid for 10 min, respectively. Following centrifugation and neutralization in double-distilled water, the tissue pellets were each inoculated onto two slopes of LJ supplemented with pyruvate and one slope of LJ with glycerol. All cultures were monitored weekly for colony growth for 10 weeks.

All tracheal lavage samples and the second set of tissue samples were cultured in the BSL3 TB laboratory at Stellenbosch University. Tissue was homogenized (Bullet blender, Next Advance, Averill Park, NY, USA) after which 5 ml BD MycoPrep^™^(Becton Dickinson, Franklin Lakes, NJ, USA) was added to each 1 cm^3^ of sample and vortexed intermittently for 15 min at 37°C. An equal amount of phosphate buffered saline (PBS) was added to samples to be neutralized. All samples were centrifuged for 15 min at 3000 x g and the supernatant decanted. Each pellet was resuspended in 1 ml PBS and 500 ul of this suspension was transferred to a Mycobacteria Growth Indicator Tube (MGIT^™^) and incubated in a BACTEC^™^ MGIT^™^ 960 Mycobacterial Detection System (both Becton Dickinson). Lavage fluid was processed in a similar manner as previously described [22]. Cultures which were Ziehl-Neelsen stain-positive were identified to species by sequencing fragments of the 16S ribosomal DNA [23] and *gyrB* genes [24].

To identify mycobacterial species, one ml of uncontaminated culture was boiled for one hour at 95°C, followed by polymerase chain reaction DNA amplification (PCR) [25]. Sequencing of the 16S rRNA gene was done using 1.1pmol/ul16S rRNA forward primer (5’—AGA GTT TGA TCC TGG CTC AG– 3’) at the Central Analytical Facility (CAF) of Stellenbosch University, South Africa [25]. Accession numbers and obtained sequences were edited and analysed using the Ribosomal Differentiation of Microorganisms (RIDOM) project (http://www.ridom-rdna.de) and the National Center for Biotechnology Information (NCBI) Blast sequence alignment tool (http://blast.ncbi.nlm.nih.gov).

### Detection of *Mycobacterium bovis* in formalin-fixed, paraffin-embedded tissue sections and/or frozen tissues by polymerase chain reaction

DNA was extracted either from formalin-fixed paraffin-embedded (FFPE) tissue sections (5 microns) or from frozen tissue homogenates. DNA extraction from FFPE tissue samples was performed using a crude boil/snap-freeze technique as previously described [26]. DNA extraction from the tissue homogenates was performed using the QIAamp^®^ DNA mini kit (Qiagen, Germany), according to the manufacturer’s instructions. Extracted DNA samples were stored at -20°C until further analysis.

PCR primers targeting the insertion sequence IS*6110* were used to test the extracted DNA samples of the three experimentally infected rhinoceroses for members of the *M*. *tuberculosis* complex [27]. The PCR master mix contained 1 unit HotStarTaq Plus DNA polymerase (Qiagen), 1x PCR buffer (containing 1.5mM MgCl2) (Qiagen), 200μM of each dNTP (Qiagen), 0.2μM of each primer (IDT), 1x Coral Load (Qiagen), 0.8μl of DNA template and nuclease-free water (Qiagen) in a total reaction volume of 20μl. DNA from *M*. *bovis* culture was used as a positive control and nuclease-free water served as a negative control. The reaction was performed on the Veriti thermal cycler (Applied Biosystems, California, USA). Initial denaturation was set at 95°C for 5 min, followed by 35 cycles of denaturation at 94°C (30 sec), annealing at 69°C (30 sec) and extension at 72°C (1 min) and final extension at 72°C for 10 min. PCR products were analysed using electrophoresis on a 2% agarose (Cleaver Scientific, England, UK) gel, stained with ethidium bromide (Invitrogen, Massachusetts, USA) and documented with the GelCompar Documentation System (Bio-Rad, California, USA).

## Results

### *Mycobacterium bovis* infective dose

The final infective doses administered were determined as 2.1 x 10^3^ cfu for animal PB1, 1.8 x 10^2^ cfu for animal PB2 and 1.4 x 10^3^ for animal PB4.

### *Mycobacterium bovis* culture and PCR

Although non-tuberculous mycobacteria (*M*. *avium*, *M*. *intracellulare*, *M*. *fortuitum*, etc.) were isolated from tracheal lavage cultures on sporadic occasions during the 24-month monitoring period (4 pre-infection, 20 post-infection), only PB1 had a confirmed *M*. *bovis* isolate from the tracheal samples collected 5 months post-infection. No mycobacteria were isolated from any of the lesions submitted for culture in either the liquid or solid phase systems at the University of Pretoria or the University of Stellenbosch, respectively.

PCR assays conducted on DNA extracted from 47 individual FFPE tissues from PB1, PB2 and PB4 yielded specific DNA products (123 bp in size) from two lesions in the left lung of PB1 and from DNA extracted from frozen right lung tissue of PB2. No DNA amplification was obtained from 7 samples of PB4, 9 pooled samples from the control rhinoceroses, nor from 17 individually extracted DNA samples from FFPE tissues from 5 control rhinoceroses.

### Clinicopathologic results

No detectable clinical signs were associated with experimental *M*. *bovis* infection in any of the rhinoceroses throughout the study period. Individuals were monitored for changes in appetite, demeanour/behavior, defecation, evidence of coughing, change in respiratory character or discharge. Similarly, there were no clinically significant changes in hematological and serum biochemical parameters in the three rhinoceroses, although each showed mild decreases in white blood cell counts. Variable decreases in eosinophil counts occurred in PB1 and PB2. All three rhinoceroses showed significant increases in albumin between the pre- and post-infection periods, with a concurrent decrease in globulins. Significant increases in alkaline phosphatase (ALP) were observed in PB1 and PB4 post-infection.

### Macro- and histopathology

Disseminated progressive tuberculosis was not found in any of the rhinoceroses examined. Lung and lymph node lesions were observed in PB1, PB2 and PB4, which varied in numbers, macroscopic and histological appearance and location. Findings are summarized in Table 1 (detailed descriptions in S1, S2 and S3 Tables).

**Table 1:** Characterization of lesions in lungs and lymph nodes of three experimentally infected white rhinoceroses.

Animal	Organ	PCR	ZN	AI	SI	SNI	CNG	FIN	FN
**PB1**	Lymph nodes	0	1^#^	3	0	0	9	0	4
**PB1**	Lung	2	0	1	6	0	16	71	30
**PB2**	Lymph nodes	0	1^#^	0	0	0	3	0	0
**PB2**	Lung	1	0	11	32	9	44	59	4
**PB4**	Lymph nodes	0	0	0	0	0	0	0	0
**PB4**	Lung	0	0	4	14	2	7	2	1

PCR, polymerase chain reaction, ZN, Ziehl-Neelsen staining, AI, acute inflammation; SI, subacute lymphoplasmacytic inflammation; SNI, subacute necrotizing inflammation; CNG, chronic necrogranuloma; CMG, chronic mineralized granuloma; FIN, fibrous nodule with inflammatory cells in the center; FN, fibrous nodule.

^#^ tracheobronchial lymph node.

#### Lymph node lesions

**Fig 2:**
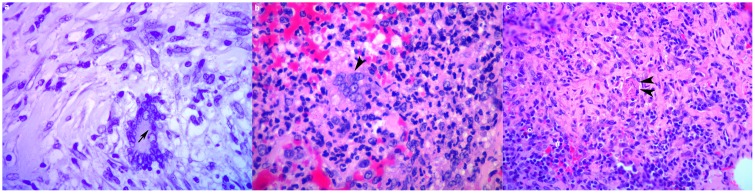
(a-c). Histological illustration of infectious agents in white rhinoceros lung. (a) A single fine short acid fast bacterial rod consistent with Mycobacteria (black arrow) in a multinucleate giant cell in a granuloma in the tracheobronchial lymph node (PB1, Ziehl Neelsen, x400). (b) A syncytial cell (black arrowhead) consistent with viral etiology associated with acute interstitial pneumonia (PB2, H&E, x400). (c) Nematode larval section (double arrowhead) associated with subacute lymphoplasmacytic (#) and eosinophilic (°) pneumonia (PB2, H&E, x100).

Macroscopically, all lymph nodes showed variable numbers of grey foci (<2mm dia.) that consisted histologically of areas of sinus histiocytosis. The trachea-bronchial lymph nodes of PB1, PB2 and PB4 were moderately enlarged due to lymphoid hyperplasia. Those of PB1 and PB2 contained multiple encapsulated calcified firm nodules (1-5mm dia.) that corresponded, histologically, to thickly encapsulated foci of variably mineralized necrotic debris surrounded by a discontinuous thin layer of epithelioid cells with small numbers of multinucleate giant cells (Fig 1d) containing rare single fine short acid fast bacterial rods consistent with mycobacteria (Fig 2a). No microscopic granulomas or other inflammatory lesions were present in sections of tracheobronchial lymph nodes from PB4. One axillary lymph node of PB1 contained multiple small (c. 250μm) medullary foci of intense eosinophilic and neutrophilic inflammation with small numbers of multinucleate giant cells. Renal lymph nodes (PB1, PB2) contained variably sized encapsulated, gritty, mottled pale tan to deep red foci consisting histologically of marked variably mineralized connective tissue mixed with haematoidin pigment and scattered clusters of haemosiderin-laden macrophages. Moderate numbers of neutrophils and lymphocytes were present in the prescapular lymph node sinuses of PB2. The deep cervical lymph nodes of PB4 contained clusters of macrophages filled with haemosiderin and haematoidin mixed with moderate numbers of eosinophils. Large numbers of eosinophils occurred in the parenchyma of the intercostal lymph node of PB4. Remaining lymph nodes showed varying degrees of lymphoid hyperplasia and sinus histiocytosis but no inflammatory lesions.

#### Lung lesions (PB1, PB2, PB4)

All rhinoceroses had large numbers of tiny firm palpable nodules (<1mm dia) in the lung (particularly the dorsocaudal lobes), that corresponded variably to histological foci of inflammation and fibrosis (S1, S2 and S3 Tables). Variable numbers of larger haemorrhagic to firm pale tan and/or gritty lesions were also present. Necrotizing encapsulated granulomatous lesions accounted for less than a quarter of the lung lesions (PB1: 14.88%; PB2: 27.49%; PB4: 23.33%); none of these contained acid fast organisms (ZN stain).

Many of the histological lesions were chronic with no indication of aetiology (PB1: 79.17%; PB2: 42.11%; PB4: 10%); chronic granulomatous inflammatory foci with mineralized centers, fibrous nodules with a few mixed inflammatory cells in the center and fibrous nodules. A lesion in the left lung lobe of PB2 consisted of a chronic mineralized necrogranuloma with small numbers of dichotomous branching septate fungal hyphae in the capsule. Mild multifocal subacute lymphoplasmacytic and histiocytic inflammation without granuloma formation was common in PB4 (PB1: 3.57%; PB2: 18.71%; PB4: 46.67%). Subacute inflammation without granulomas but with necrotic cellular and inflammatory debris present were rare (PB1: 0%; PB2: 5.26%; PB4: 6.67%). In PB2 one focus of subacute necrotizing inflammation was associated with a suspected cestode cyst. Mild acute multifocal, variably hemorrhagic or necrotizing, neutrophilic and/or eosinophilic interstitial and bronchopneumonia were relatively rare (PB1: 2.38%; PB2: 6.43%; PB4:13.33%). Several acute inflammation foci in PB2 and PB4 respectively were associated with syncytial cells (Fig 2b). In PB2 a focus of acute inflammation was associated with a fragment of suspected nematode larva in left lung lesion nine with eosinophils and multinucleate giant cells (Fig 2c). Lung lesions were often adjacent to airways, but only in a left lung lobe lesion in PB1 and in areas of acute pneumonia in PB2 and PB4, where the bronchiolar epithelium breached by an inflammatory reaction.

#### Significant lesions in other tissues (PB1, PB2, PB4)

There were moderate numbers of firm gritty flat pale yellow nodules on the liver capsule and near the hilus in the hepatic parenchyma (<5x3x3mm) of PB1, consisting of CNGs. Mild multifocal eosinophilic and granulomatous gastritis in PB1 was associated with a nematode cross-section. PB2 had a number of inflammatory lesions not seen in the other animals: focal chronic ulcerative neutrophilic and eosinophilic dermatitis (20x12mm) on the left mandible and nasal epithelium; moderate acute necrohemorrhagic neutrophilic pharyngitis as well as multifocal acute perivascular cerebral and meningeal hemorrhage. Mild multifocal crescentic to global membranoproliferative glomerulonephritis with mild glomerulosclerosis was also present.

#### Lung and lymph node lesions (control animals)

Culture of a tracheal lavage done three years prior to death from a 15 year old male was negative. Culture of a tracheal lavage wash done two years prior to death was negative in a 3–5 year old male that had mild acute eosinophilic interstitial pneumonia at death. Pulmonary CNGs in an adult female occurred in and around bronchioles and contained large numbers of eosinophils. Lung from an elderly male contained two irregular foci of chronic interstitial inflammation associated with pale pink to brown pigment and small amounts of bright eosinophilic filamentous material (possibly helminth cuticular material). Two hepatic CNG lesions with abundant eosinophils were also present. A large CNG in the left CdL lung contained a cross section of a nematode in the center.

## Discussion

The susceptibility of the white rhinoceros to *M*. *bovis* was confirmed by successful experimental infection based on the presence of acid-fast organisms, necrotizing granulomatous lesions in the tracheobronchial lymph nodes of PB1 and 2, ante mortem isolation of *M*. *bovis* from the respiratory tract of PB1, and detection of *M*. *bovis* genetic material by PCR in the lungs of PB1 and PB2. However, infection appeared to be contained based on clinical, pathological, and microbiological findings, suggesting that healthy young white rhinoceros may be resistant to the development of tuberculosis disease.

Isolation of *M*. *bovis* from otherwise sterile sites in the host serves as evidence of BTB infection which is the result of successful colonization and penetration of target tissues, a moderate degree of multiplication and evasion of the host’s immune responses. Recovery of viable *M*. *bovis* from a bronchial wash from PB1 five months post-infection therefore demonstrated active infection. Likewise, the presence of acid-fast bacilli in a few thoracic lymph node lesions as well as *M*. *bovis* DNA in the lungs of PB1 and PB2 convincingly demonstrated the invasion, mild tissue damage and limited dissemination of *M*. *bovis* in tissues of the respiratory tract as part of the infection process. However, and most importantly, no viable *M*. *bovis* could be isolated (using two different culture systems) 20 months post-infection, indicating that the infection in those tissues was successfully contained and most probably sterilized during this period. Histological lesions evaluated 20 months post-infection were consistent with chronic and contained infection foci supporting the culture results. In contrast, progressive disseminated disease which is defined as a state in which the functions of part of or the whole body are disrupted, was absent, both clinically and pathologically. These findings suggest that healthy young white rhinoceroses may possess a fair level of resistance to disease during a single infection with *M*. *bovis* by controlling or eliminating infection.

The observation that PB4 showed the least pathological changes of the three infected rhinoceroses and *M*. *bovis* was not detected by either culture or PCR may indicate that this animal was possibly more resistant to infection. An evaluation of the immune response profiles of all three rhinoceroses during the study is underway and may provide more insight.

White rhinoceroses are not the only perissodactyls for which resistance to tuberculosis has been noted [5]. In domestic horses, tuberculosis was not uncommon and described as a potentially malicious condition in Europe before the disease was successfully controlled in cattle, but it was never considered of epidemiological significance (reviewed by O’Reilly and Daborn) [28]. Since that time, equine TB cases have become increasingly rare [29]. Clinical signs including coughing were recently reported in a horse with *M*. *bovis* infection which resolved within three months without relapse during the subsequent eight years. This suggests that although horses may contract and harbour *M*. *bovis*, infection may be more easily contained and possibly eliminated [30]. In tapirs, tuberculosis caused by *M*. *tuberculosis* has been reported in both captive Brazilian and Malayan tapirs which appeared to contract the infection through inter-species rather than intra-species transmission [31, 32]. These results are similar to our findings in the experimentally infected rhinoceros and support the view that perissodactyl species are more resistant to tuberculosis than many other species. This does, however, not rule out the possibility of severe and generalised tuberculosis disease in individual cases, given the enabling circumstances of high infection pressure (i.e. endemic areas) leading to opportunities of recurrent exposure and potentially aggravated by conditions of increased susceptibility of the host, e.g. immunosuppression or concurrent infections. Since the white rhinoceroses in this study were habituated to captive management prior to infection, it is likely that they experienced minimal stressors that could alter host defenses.

Detailed histological descriptions of tuberculosis in rhinoceroses are limited, but the lesions seen in this study were similar to those described in an incidental finding in a captive black rhinoceros with BTB infection [8], most likely acquired prior to translocation from a free-range environment in South Africa [33]. This suggests that the infectious doses used in this report were at least *en par* with or higher than natural exposure levels. In experimental *M*. *bovis* infection studies in other species very similar infectious doses were reported to cause lesions compatible with those observed in natural infection. Experimental infection of African buffalo using the same M. bovis genotype resident in the KNP at a dose of 320 colony forming units caused gross pathology in 4 out of 11 buffalo and culture positive status in 6 out of 11 buffalo [34]. Similar results were obtained following experimental infection of deer with 500 colony forming units via the tonsilar route [35]. The effective infectious dose in these two models may, however, have been slightly lower than reported due to the delivery of the inoculum into and the associated potential for spillage from the tonsilar crypt as opposed to the controlled delivery into both mainstem bronchi in the rhinoceroses.

The fact that *M*. *bovis* was isolated from the black rhinoceros but not from PB1, PB2 and PB4 at post- mortem may suggest reactivation of a latent infection in the black rhinoceros due to changes in immune status. The black rhinoceros had suffered from chronic diarrhea and general compromised health for some time before euthanasia. The possibility of latent infection has also been discussed for horses, as Francis (1958) suggested that foals become infected with *M*. *bovis* via milk but only succumb to clinical disease during adulthood (cited by O’Reilly and Daborn) [28].

Latent infections in humans are well described, in which there is a persistent response to mycobacterial infection but without clinically active disease [36]. Reactivation risk is estimated to be 5–10%, with most cases occurring in the first 5 years. Predisposing factors such as conditions causing immunocompromised, concurrent infections, being a member of a high TB prevalence population, malnutrition, and age may lead to disease progression [36]. Similar risk factors may apply to animals with asymptomatic infection, in which a minority could have latent infection which may lead to reactivation years later. The lesions observed in our rhinoceros were similar to those described for latent TB in Asian elephants [37]. Therefore, it is possible that PB1 or PB2 might have developed latent infection; however, long-term studies would be required to evaluate outcomes in this species.

Clinicopathologic changes have been reported in humans and animals with tuberculosis. Anaemia, leucocytosis with neutrophilia, lymphopenia, and monocytosis; hypoalbuminemia, elevated alkaline phosphatase, lactic dehydrogenase, and aspartic transaminase have been reported in human patients with severe pulmonary tuberculosis [38]. However, these responses are nonspecific and variably observed, especially in patients without advanced disease. In one study, 71% of sputum-positive TB patients had normal white blood cell counts [39]. Due to the scarcity of TB cases in rhinoceroses, there is limited information on changes in hematologic and biochemical values in these species. Nonspecific changes including anaemia, mild leucocytosis, elevated globulins, and BUN have been observed in clinically affected black rhinoceroses. In this study, observed differences in hematological and serum biochemistry values post-infection were limited to increases in albumin (all 3 rhinoceroses) and ALP (PB1 and PB4), mildly decreased total white blood cell count, and decreased eosinophil counts in PB1 and PB2. Since the observed changes in the infected rhinoceroses were also seen in uninfected animals, the hematological changes in the infected rhinoceroses were probably unrelated to *M*. *bovis* infection.

Hypoalbuminemia has been used as a predictive risk factor for mortality in human TB patients as a reflection of nutritional status and disease chronicity [40]. Increased albumin values were observed in the experimentally infected rhinoceroses, however, albumin and total protein values increased significantly in uninfected boma adapted rhinoceroses [20], therefore these changes were probably unrelated to *M*. *bovis* infection. The increases in ALP observed in PB1 and PB4 are suggestive of hepatic disease, as reported in human TB patients [38]. Although CNG were present in the liver, there was no indication that *M*. *bovis* was the underlying etiology. However, these increases were statistically but not clinically significant. Hypercalcemia has also been associated with granulomatous disease such as tuberculosis [41]. However, no changes in calcium, phosphorus or BUN were observed in the experimentally infected rhinoceroses. Therefore, the lack of a discernible clinical response and minimal hematological and biochemical changes associated with the experimental infection suggest that white rhinoceroses are capable of containing early *M*. *bovis* infection which is consistent with the rare reports of BTB in this species. We also conclude that hematological and serum biochemistry changes are insensitive markers for diagnosis of early *M*. *bovis* infection in white rhinoceros but may provide useful information pertaining to general health status.

Post mortem examinations of white rhinoceroses which died from natural causes or as a consequence of poaching incidents have been performed by veterinary personnel in the KNP and HiP where BTB is endemic in the buffalo populations. The data collected during these investigations can contribute meaningfully towards determining the risk profile of white rhinoceroses in terms of BTB. No macropathological signs consistent with BTB have been reported in rhinoceros examined between 2008 and the time of this study. This does not rule out the potential presence of small localized and contained lesions which could have been easily missed on macroscopic inspection.

Histologically, none of the various inflammatory or granulomatous lung lesions could be definitely ascribed to tuberculosis due to the paucity of acid fast organisms and chronic resolved nature of the lesions. Other possible causes of granulomatous lesions, in addition to BTB, include inhaled foreign material, fungi and helminths, evidence of all of which were found in the experimental as well as the control rhinoceroses. The various acute inflammation lesions in PB2 and PB4 more closely resembled viral lesions than BTB, given the presence of syncytial cells. The lungs of free-ranging control rhinoceroses commonly contained small to large numbers of inflammatory lesions including granulomas. This may be a feature of the feeding habits of rhinoceroses that graze close to the ground [42] and may regularly inhale foreign and infectious material including mycobacteria from contaminated environmental sources.

This experimental infection study was a key step to advance our understanding of the susceptibility, pathology and risk profile of white rhinoceroses with regard to BTB during a period of 20 months after infection. Prior to this study, there have only been two confirmed *M*. *bovis* infected black rhinoceros in South Africa [43]. Therefore, it was not possible to investigate clinical, pathological, and immunological changes associated with BTB in naturally infected rhinoceros. It was considered ethically acceptable to experimentally infect three male white rhinoceros to address the pressing concerns of BTB on conservation of this near threatened species (www.iucnredlist.org/pdflink.16980466). White rhinoceros can be legally hunted and privately owned in South Africa, and males are often considered surplus to the population. In addition, the discovery of *M*. *bovis* infected white and black rhinoceros in KNP in 2016 (P. Buss, pers. comm.), during the prolonged drought, has provided a critical impetus for understanding the risk, pathological and immunological responses of rhinoceros to BTB. The pre- and post mortem culture and histological findings from this study were able to provide the first data illustrating the comparatively low risk of progression of *M*. *bovis* infection and shedding by healthy white rhinoceroses following a single exposure. This is further supported by the absence of any observations indicative of BTB during field post-mortem examinations of white rhinoceroses which had died from either natural causes or poaching related events in the KNP to date. Therefore epidemiological studies were not an available alternative to the experimental infection study. Additionally, the results of this study are important as they provide robust data of the temporal changes in clinical and immunological responses in experimentally infected individual rhinoceros (described in a separate paper). However, for a more comprehensive understanding of the long term impact of BTB on the health of white rhinoceroses, a longitudinal study would be required.

## Conclusion

Healthy white rhinoceroses were susceptible to infection with *M*. *bovis* but did not develop progressive disease following a single exposure. Clinical signs were absent, significant hematological and biochemical changes, and pathological lesions consistent with BTB were minimal, and could be due to causes other than *M*. *bovis* infection. The infection appeared to be contained. The lack of isolation of viable organisms post-mortem and isolation from only one sample ante-mortem strongly indicate that the risk of transmission or progression to disseminated disease is low in otherwise healthy rhinoceroses.

## Supporting information

S1 Table Macroscopic, culture, molecular, and histological findings in experimentally infected rhinoceros PB1. (XLSX)

S2 Table Macroscopic, culture, molecular, and histological findings in experimentally infected rhinoceros PB2. (XLSX)

S3 Table Macroscopic, culture, molecular, and histological findings in experimentally infected rhinoceros PB4. (XLSX)

S4 Table Macroscopic, culture, molecular, and histological findings in 11 control rhinoceroses. (XLSX)

## References

[1] Bengis RG. Tuberculosis in Free-Ranging Mammals In: Fowler M.E. & Miller R.E., editor. Zoo and Wild Animal Medicine. Philadelphia, Pennsylvania: W.B. Saunders Company; 1999 pp. 101–114.

[2] Cousins DV, Huchzermeyer HF, Griffin JF, Brueckner GK, van Rensburg IBJ, Kriek NPJ. Tuberculosis In: Infectious diseases of livestock. Cape Town: Oxford University Press; 2004.

[3] Renwick AR, White PC, Bengis RG. Bovine tuberculosis in southern African wildlife: a multi-species host-pathogen system. Epidemiol Infect. 2007;135: 529–540. doi: 10.1017/S0950268806007205 16959052 10.1017/S0950268806007205 PMC2870607

[4] Pesciaroli M, Alvarez J, Boniotti MB, Cagiola M, Di Marco V, Marianelli C, et al Tuberculosis in domestic animal species. Res Vet Sci. 2014;97: S78–S85. doi: 10.1016/j.rvsc.2014.05.015 25151859 10.1016/j.rvsc.2014.05.015

[5] Miller M, Lyashchenko K. Mycobacterial Infections in Other Zoo Animals In: Mukundan H, Chambers MA, Waters WR, Larsen MH, editors. Tuberculosis, Leprosy and Mycobacterial Diseases of Man and Animals: The Many Hosts of Mycobacteria. Boston, Massachusetts: CAB International; 2015 pp. 277–295.

[6] Stetter MD, Mikota SK, Gutter AF, Monterroso ER, Dalovisio JR, Degraw C, et al Epizootic of *Mycobacterium bovis* in a zoologic park. J Am Vet Med Assoc. 1995;207: 1618–1621. 7493904

[7] Miller M, Michel A, van Helden P, Buss P. Tuberculosis in rhinoceros: an underrecognized threat? Transbound Emerg Dis. 2016;doi: 10.1111/tbed.12489 26996520 10.1111/tbed.12489

[8] Espie IW, Hlokwe TM, Pittius NCG, Lane E, Tordiffe ASW, Michel AL, et al Pulmonary infection due to *Mycobacterium bovis* in a black rhinoceros (Diceros bicornis minor) in South Africa. J Wildl Dis. 2009;45: 1187–1193. doi: 10.7589/0090-3558-45.4.1187 19901395 10.7589/0090-3558-45.4.1187

[9] Michel AL, Bengis RG, Keet DF, Hofmeyr M, de Klerk LM, Cross PC, et al Wildlife tuberculosis in South African conservation areas: implications and challenges. Vet Microbiol. 2006;112: 91–100. doi: 10.1016/j.vetmic.2005.11.035 16343819 10.1016/j.vetmic.2005.11.035

[10] De Klerk L, Michel AL, Bengis RG, Kriek NPJ, Godfroid J. BCG vaccination failed to protect yearling African buffaloes (Syncerus caffer) against experimental intratonsilar challenge with *Mycobacterium bovis*. Vet Immunol Immunopath. 2010;137: 84–92. 10.1016/j.vetimm.2010.04.013 20684850

[11] Michel AL, de Klerk LM, Buss P, Hofmeyr M, Cooper D, Bengis R. Tuberculosis in South African Wildlife: Lions, African Buffalo and Other Species In: Mukundan H, Chambers M, Waters R, Larsen M.H., editors. Tuberculosis, Leprosy and Mycobacterial Diseases of Man and Animals. Boston, USA: CAB International; 2015 pp. 365–385.

[12] Duncan AE, Lyashchenko K, Greenwald R, Miller M, Ball R. Application of elephant TB Stat-Pak assay and MAPIA (Multi-Antigen Print Immunoassay) for detection of tuberculosis and monitoring of treatment in black rhinoceros (Diceros bicornis). J Zoo Wildl Med. 2009;40: 781–785. doi: 10.1638/2009-0044.1 20063826 10.1638/2009-0044.1

[13] Miller MA, Greenwald R, Lyashchenko KP. Potential for serodiagnosis of tuberculosis in black rhinoceros (Diceros bicornis). J Zoo Wildl Med. 2015;46: 100–104. doi: 10.1638/2014-0172R1.1 25831581 10.1638/2014-0172R1.1

[14] Morar D, Tijhaar E, Negrea A, Hendriks J, van Haarlem D, Godfroid J, et al Cloning, sequencing and expression of white rhinoceros (Ceratotherium simum) interferon-gamma (IFN-gamma) and the production of rhinoceros IFN-gamma specific antibodies. Vet Immunol Immunopath. 2007;115: 146–154. 10.1016/j.vetimm.2006.10.016 17118460

[15] Morar D, Schreuder J, Mény M, van Kooten PJS, Tijhaar E, Michel AL, et al Towards establishing a rhinoceros-specific interferon-gamma (IFN-γ) assay for diagnosis of tuberculosis. Transbound Emerg Dis. 2013;60 Suppl 1: 60–66. 24171850 10.1111/tbed.12132

[16] Miller M, Kruger M, Kruger M, Olea-Popelka F, Buss P. A scoring system to improve decision making and outcomes in the adaptation of recently captured white rhinoceroses (Ceratotherium simum) to captivity. J Wildl Dis. 2016;52: S78–S85. doi: 10.7589/52.2S.S85 26845302 10.7589/52.2S.S85

[17] Hlokwe TM, van Helden P, Michel AL. Evidence of increasing intra and inter-species transmission of *Mycobacterium bovis* in South Africa: Are we losing the battle? Prev Vet Med. 2014;115: 10–17. doi: 10.1016/j.prevetmed.2014.03.011 24703246 10.1016/j.prevetmed.2014.03.011

[18] De Klerk L, Michel AL, Grobler DG, Bengis RG, Bush M, Kriek NP, et al An experimental intratonsilar infection model for bovine tuberculosis in African buffaloes, Syncerus caffer. Onderstepoort J Vet Res. 2006;73: 293–303. 17283730

[19] Miller M, Buss P, Wanty R, Parsons S, van Helden P, Olea-Popelka F. Baseline hematologic results for free-ranging white rhinoceros (Ceratotherium simum) in Kruger National Park, South Africa. J Wildl Dis. 2015;51:916–922. doi: 10.7589/2015-03-081 26267457 10.7589/2015-03-081

[20] Mathebula N, Miller M, Buss P, Joubert J, Martin L, Kruger M, et al Biochemical values in free-ranging white rhinoceros (Ceratotherium simum) in Kruger National Park, South Africa. J Zoo Wildl Med. 2012;43: 530–538. doi: 10.1638/2011-0259R.1 23082517 10.1638/2011-0259R.1

[21] Bancroft JD, Gamble M. Theory and practice of histological techniques. Elsevier Health Sciences, 2008.

[22] Miller M, Buss P, Hofmeyr J, Olea-Popelka F, Parsons S, van Helden P. Antemortem diagnosis of *Mycobacterium bovis* infection in free-ranging African lions (Panthera leo) and implications for transmission. J Wildl Dis. 2015;51: 493–497. doi: 10.7589/2014-07-170 25647595 10.7589/2014-07-170

[23] Harmsen D, Dostal S, Roth A, Niemann S, Rothgänger J, Sammeth M, Albert J, Frosch M, Richter E. RIDOM: comprehensive and public sequence database for identification of Mycobacterium species. BMC Infect Dis. 2003;3:1. 14611664 10.1186/1471-2334-3-26 PMC280682

[24] Huard RC, Fabre M, de Haas P, Lazzarini LC, van Soolingen D, Cousins D, et al Novel genetic polymorphisms that further delineate the phylogeny of the *Mycobacterium tuberculosis* complex. J Bacteriol. 2006;188: 4271–4287. doi: 10.1128/JB.01783-05 16740934 10.1128/JB.01783-05 PMC1482959

[25] Katale BZ, Mbugi EV, Siame KK, Keyyu JD, Kendall S, Kazwala RR, et al Isolation and potential for transmission of *Mycobacterium bovis* at human-livestock-wildlife interface of the Serengeti ecosystem, Northern Tanzania. Transbound Emerg Dis. 2015;10. 10.1111/tbed.12445 PMC5434928 26563417

[26] Miller J, Jenny A, Rhyan J, Saari D, Suarez D. Detection of *Mycobacterium bovis* in formalin-fixed paraffin-embedded tissues of cattle and elk by PCR amplification of an IS6110 sequence specific for *Mycobacterium tuberculosis* complex organisms. J Vet Diag Invest. 1997 9:244–249. 10.1177/104063879700900304 9249162

[27] Eisenach KD, Cave MD, Bates JH, Crawford JT. Polymerase chain reaction amplification of a repetitive DNA sequence specific for *Mycobacterium tuberculosis*. J Infect Dis. 1990;161: 977–981. 2109022 10.1093/infdis/161.5.977

[28] O'Reilly LM, Daborn CJ. The epidemiology of *Mycobacterium bovis* infections in animals and man: A review. Tubercle Lung Dis. 1995;76: 1–46. 10.1016/0962-8479(95)90591-x 7579326

[29] Pavlik I, Jahn P, Dvorska L, Bartos M, Novotny L, Halouzka R. Mycobacterial infections in horses: A review of the literature. Vet Med. 2004;49: 427–440.

[30] Hlokwe TM, Sutton D, Page P, Michel AL. Isolation and molecular caracterization of *Mycobacterium bovis* causing pumonary tuberculosis and epistaxis in a thorughbred horse. BMC Vet Res. 2016;12:179 doi: 10.1186/s12917-016-0813-6 27590011 10.1186/s12917-016-0813-6 PMC5010722

[31] Murakami PS, Monego F, Ho JL, Gibson A, Javorouski ML, Bonat M, et al Detection of RDRIO strain of *Mycobacterium tuberculosis* in tapirs (Tapirus terrestris) from a zoo in Brazil. Arquivo Brasileiro de Medicina Veterinaria e Zootecnia. 2012;43: 872–875. 10.1638/2010-0108R.1 23272356

[32] Michel AL, Venter L, Espie IW, Coetzee ML. *Mycobacterium tuberculosis* infections in eight species at the National Zoological Gardens of South Africa, 1991–2001. J Zoo Wildl Med. 2003;34: 364–370. doi: 10.1638/02-063 15077712 10.1638/02-063

[33] Michel AL, Musoke J, Hlokwe TM. Spillover, spillback and translocation of bovine tuberculosis in South Africa. 12th SAVEPM conference, Port Elizabeth. 21–23 August 2014.

[34] De Klerk L, Michel AL, Grobler DG, Bengis RG, Bush M, Kriek NPJ, Hofmeyr MS, Griffin JFT, Mackintosh CG. 2006 An experimental intratonsilar infection model for bovine tuberculosis in African buffalo (Syncerus caffer). Onderstepoort J Vet Res. 2006;73:293–303. 17283730

[35] Griffin JFT, Mackintosh CG, Buchan GS. Animal models of protective immunity in tuberculosis to evaluate candidate vaccines. Trends Microbiol. 1995;3:418–422. 8574514 10.1016/s0966-842x(00)88994-5

[36] Ai JW, Ruan Ql, Liu QH, Zhang WH. Updates on the risk factors for latent tuberculosis reactivation and their managements. Emerg Microbes Inf. 2016;doi: 10.1038/emi.2016.10 26839146 10.1038/emi.2016.10 PMC4777925

[37] Landolfi JA, Terio KA, Miller M, Junecko BF, Reinhart T. Pulmonary tuberculosis in Asian elephants (Elephas maximus): histologic lesions with correlation to local immune responses. Vet Pathol. 2015;52: 535–542. doi: 10.1177/0300985814548517 25228055 10.1177/0300985814548517

[38] Morris CDW, Bird AR, Nell H. The haematological and biochemical changes in severe pulmonary tuberculosis. QJM. 1989;73: 1151–1159. 2616737

[39] Yaranal PJ, Umashankar T, Harish SG. Hematological profile in pulmonary tuberculosis. Int J Health Rehabil Sci. 2013;2: 50–55.

[40] Okamura K, Nagata N, Wakamatsu K, Yonemoto K, Ikegame S, Kajiki A, et al Hypoalbuminemia and lymphocytopenia are predictive risk factors for in-hospital mortality in patients with tuberculosis. Intern Med. 2013;52: 439–444. 23411698 10.2169/internalmedicine.52.8158

[41] Roussos A, Lagogianni I, Gonis A, Ilias I, Kazi D, Patsopoulos D, et al Hypercalcaemia in Greek patients with tuberculosis before the initiation of anti-tuberculosis treatment. Respir Med. 2001;95: 187–190. doi: 10.1053/rmed.2000.1019 11266235 10.1053/rmed.2000.1019

[42] Perrin MR, Brereton-Stiles R. Habitat use and feeding behaviour of the buffalo and the white rhinoceros in the Hluhluwe-Umfolozi Game Reserve. S Afr J Wildl Res. 1999;29: 72–80.

[43] Miller MA, Buss PE, van Helden PD, Parsons SD. *Mycobacterium bovis* in a free-ranging black rhinoceros, Kruger National Park, South Africa, 2016. Emerg Infect Dis. 2017; 23(3):557–558. doi: 10.3201/eid2303.161622 28221132 10.3201/eid2303.161622 PMC5382732

